# Depression of home cage wheel running: a reliable and clinically relevant method to assess migraine pain in rats

**DOI:** 10.1186/s10194-017-0721-6

**Published:** 2017-01-13

**Authors:** Ram Kandasamy, Andrea T. Lee, Michael M. Morgan

**Affiliations:** 1 Graduate Program in Neuroscience, Washington State University, Pullman, WA USA; 2 Department of Psychology, Washington State University Vancouver, Vancouver, WA USA; 3 Washington State University Vancouver, 14204 NE Salmon Creek Ave, Vancouver, WA 98686 USA

**Keywords:** TRPA1, Headache, Voluntary activity, Nociception, Pain-depressed behavior

## Abstract

**Background:**

The development of new anti-migraine treatments is limited by the difficulty inassessing migraine pain in laboratory animals. Depression of activity is one of the few diagnostic criteria formigraine that can be mimicked in rats. The goal of the present study was to test the hypothesis thatdepression of home cage wheel running is a reliable and clinically relevant method to assess migraine painin rats.

**Methods:**

Adult female rats were implanted with a cannula to inject allyl isothiocyanate (AITC) onto the dura to induce migraine pain, as has been shown before. Rats recovered from implantation surgery for 8 days in cages containing a running wheel. Home cage wheel running was recorded 23 h a day. AITC and the migraine medication sumatriptan were administered in the hour prior to onset of the dark phase.

**Results:**

Administration of AITC caused a concentration-dependent decrease in wheel running that lasted 3 h. The duration and magnitude of AITC-induced depression of wheel running was consistent following three repeated injections spaced 48 h apart. Administration of sumatriptan attenuated AITC-induced depressionof wheel running when a large dose (1 mg/kg) was administered immediately following AITC administration. Wheel running patterns did not change when sumatriptan was given to naïve rats.

**Conclusions:**

These data indicate that home cage wheel running is a sensitive, reliable, and clinically relevant method to assess migraine pain in the rat.

## Background

Migraine is characterized by severe pain and heightened sensitivity to sensory stimuli that results in depression of normal daily activities. Migraine is difficult to study in laboratory animals because pain occurs in the absence of tissue injury and because of the limitations of existing behavioral assays [27]. Most preclinical studies of migraine assess periorbital and/or hindpaw allodynia as the dependent measure for migraine pain. Although migraine causes allodynia in a subset of migraineurs, allodynia is not a diagnostic criterion [12]. Moreover, allodynia is considered a marker of migraine progression [5, 16], rarely assessed clinically [19], and may outlast the headache and be present during interictal periods [1]. Thus, the development of better ways to assess migraine pain in laboratory animals would significantly advance migraine research.

In contrast to allodynia, the reduction in routine physical activity caused by migraine is a diagnostic criterion that is easy to replicate in laboratory animals. A number of studies have used depression of activity (e.g., locomotor activity, feeding, rearing) to assess pain resulting from headache in humans [18] and rodents [7, 9, 17, 20, 28]. The problem with these studies is that assessment was limited to 60 min or less making it impossible to quantify the full duration and magnitude of the migraine. In humans, the time course for the headache phase of migraine can last from 4 to 72 h.

We have recently shown that home cage wheel running is a sensitive and objective method to assess the magnitude and duration of chronic inflammatory pain [14]. Home cage wheel running is an especially good model of human activity because it is a voluntary behavior that shows clear diurnal rhythms that can be continuously and objectively quantified in the rat in a stress-free environment [14]. The primary goal of the present study was to test the hypothesis that home cage wheel running is a sensitive, reliable, and clinically relevant method to assess the duration and magnitude of migraine pain in laboratory rats.

Although a number of different animal models of migraine have been developed (e.g., dural inflammatory soup, systemic nitroglycerin), we used microinjection of the TRPA1 agonist allyl isothiocyanate (AITC) onto the dura to generate migraine pain. Similar to inflammatory soup, AITC is a simple and direct method to activate the dural afferents responsible for headache pain. Moreover, activation of TRPA1 degranulates mast cells to release histamine and serotonin [10] – all of which are key components of migraine pathophysiology [2]. TRPA1 receptors have also been shown to contribute to migraine in both humans and rodents [9, 21]. The clinical relevance of home cage wheel running as a method to assess migraine will be evaluated by determining whether AITC produces a concentration-dependent depression of wheel running that can be reversed by the migraine treatment sumatriptan. Reliability will be assessed by measuring the consistency of AITC to depress home cage wheel running following injections on different days. All of the experiments were conducted in female rats to be consistent with the much higher rates of migraine in women than men [25, 26].

## Methods

### Subjects

Data were collected from 64 adult female Sprague-Dawley rats bred at Washington State University Vancouver (Vancouver, WA, USA). All rats were 50–70 days old at the start of the study and randomly assigned to treatment groups. A within-subjects design was used when possible to reduce the number of animals needed. All procedures were approved by the Washington State University Animal Care and Use Committee and conducted in accordance with the International Association for the Study of Pain’s Policies on the Use of Animals in Research.

### Surgery

Prior to surgery, rats were housed in pairs in a 22–24 °C colony room on a 12/12-hour light/dark cycle (lights off at 1800 h). Animals were anesthetized with pentobarbital (50 mg/kg, i.p.) and implanted with a guide cannula (18 gauge; 4 mm long) aimed above the dura mater (AP: +1.0 mm; ML: +1.0 mm; DV: 0.8 mm). Loctite® super glue (Henkel AG & Company, KGaA, Düsseldorf, Germany) was used to form a tight seal around the guide cannula and skull, and then dental cement was used to anchor the guide cannula to two screws in the skull. Rats were maintained under a heat lamp until awake. Following surgery, each rat was housed individually in an extra tall cage (36 × 24 × 40 cm) with a running wheel. The rat was allowed to recover for 8 days following surgery in a sound-attenuating booth (2.1 × 2.2 m; Industrial Acoustics Company, Inc., Bronx, NY, USA). Food and water were available *ad libitum*.

### Running wheel

A Kaytee Run-Around Giant Exercise Wheel (Kaytee Products, Inc., Chilton, WI, USA) with a diameter of 27.9 cm was suspended from the top of the rat’s home cage. The floor of the cage was covered with cellulose bedding (BioFresh™, Ferndale, WA, USA). A thin aluminum plate (0.8 mm × 5.08 cm × 3.81 cm; K&S Precision Metals, Chicago, IL, USA) was attached to one spoke of the running wheel to interrupt a photobeam projecting across the cage with each rotation. The beam was set 18 cm above the floor of the cage so that only the rotation of the wheel, not the normal activity of the rat, would interrupt the beam. The number of wheel revolutions were summed over 5 min bins for 23 h each day using Multi-Varimex software (Columbus Instruments, Columbus, OH, USA) beginning at 1700 h. The dark phase of the light cycle when rats are most active began at 1800 h. A full description of the running wheel with video is available in our previous publication [14].

### Drugs

Allyl isothiocyanate (AITC; Sigma-Aldrich, Inc., St. Louis, MO, USA) was mixed in mineral oil at concentrations of 1 and 10% and injected into the periosteal space in a volume of 10 μL. Sumatriptan succinate (Sigma-Aldrich, Inc., St. Louis, MO, USA) was dissolved in saline (Hospira Inc, Lake Forest, IL, USA) and injected subcutaneously at doses of 0.1 and 1.0 mg/kg in a volume of 1 mL/kg. All drugs were made fresh on the day it was injected.

### Baseline acquisition

Rats were allowed unrestricted access to the wheel for 23 h/day for 8 days following surgery and prior to induction of migraine pain. The number of wheel revolutions that occurred during the 23 h prior to the first dural injection was used as the baseline activity level. Rats that ran less than 400 revolutions on the baseline day (*n* = 7 out of 71) were not included in further testing [14].

### Experiment 1: AITC concentration-response

If wheel running is a clinically relevant measure of AITC-induced migraine pain, then the duration and magnitude of depressed wheel running should depend on the intensity of the headache. This hypothesis was tested by measuring depression of wheel running to different concentrations of AITC. Following baseline testing on Day 8, the rat was injected with 10 μL of 1% AITC, 10% AITC, saline, or mineral oil onto the dura mater using an injection cannula inserted into the guide cannula. All injections were complete by 1650 h. The rats were returned to their home cage and wheel running activity recorded for the next 23 h beginning at 1700 h. This procedure was repeated every other day with the drugs administered in a counterbalanced manner, although no rat was treated in more than three conditions. Rats were euthanized 48 h after the last injection.

### Experiment 2: Repeated 10% AITC injections

If depression of wheel running is a reliable measure of migraine pain, then the magnitude and duration of AITC-induced depression of wheel running should be consistent with repeated administration. This hypothesis was tested by measuring wheel running following injection of 10% AITC onto the dura every other day until the rat had received three injections. Surgical implantation of the cannula, baseline testing, and timing of the AITC injection was identical to Experiment 1.

### Experiment 3: Sumatriptan efficacy against AITC-induced pain

If depression of wheel running is a clinically relevant measure of migraine pain, then the anti-migraine medication sumatriptan should attenuate AITC-induced depression of wheel running. This hypothesis was tested by injecting sumatriptan (0.1 mg/kg and 1 mg/kg, s.c.) or saline either 1 or 90 min post-AITC injection. Human data demonstrates that sumatriptan is only effective if administered soon after migraine onset [8]. In order to administer sumatriptan 90 min after AITC administration, animals were removed from their home cages at 1500 h, injected with AITC, and returned to their home cage. Rats were removed again 90 min later, injected with sumatriptan (1 mg/kg, s.c.) or saline, and returned to their home cage at approximately 1650 h. Wheel running was measured for 23 h beginning at 1700 h. A within-subjects, counterbalanced design was used so that each rat was tested with AITC three times. Saline was administered on one occasion and sumatriptan on the other two. Some rats were tested with different doses of sumatriptan while other rats were tested at different times (1 or 90 min). Two days separated each dural injection.

### Experiment 4: Sumatriptan effects on wheel running

In the absence of migraine pain, sumatriptan should have no effect on wheel running. This hypothesis was tested by injecting sumatriptan into naïve rats following 8 days of habituation to wheel running. These rats had no surgery and were not treated with AITC. Rats were removed from their home cages and injected with sumatriptan (1 mg/kg, s.c.) or saline and returned to their home cages at approximately 1650 h. Wheel running was measured for 23 h beginning at 1700 h. A within-subjects, counterbalanced design was used so that each rat received both saline and sumatriptan injections. Two days separated these injections.

### Data analysis

Baseline activity was defined as the total number of wheel revolutions during the 23 h preceding the first injection. An average hourly nighttime running rate was used as the baseline for hour-by-hour analysis. Given individual differences in wheel running, subsequent wheel running data are presented as a percent change from each rat’s baseline value. All data are expressed as mean ± SEM. Nearly all running occurs during the dark phase of the light cycle [14], so only data collected during the dark phase and the one hour preceding the dark phase were analyzed. Percentage of baseline running was averaged over the 3-hour period following injection of AITC in order to compare the magnitude of migraine-pain induced depression of wheel running. Data were analyzed with an independent samples t-test or one-way ANOVA. Because animals whose guide cannulas were defective (*n* = 6) and whose wheels malfunctioned (*n* = 6) were not available for all of the within-subjects conditions, groups were treated as independent samples. Statistical significance was defined as a probability of < 0.05.

## Results

### Experiment 1: AITC concentration-response

**Fig. 1:**
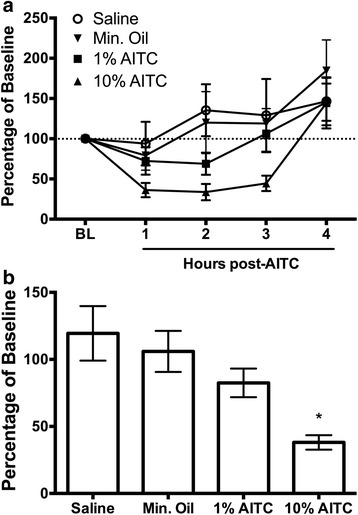
Dural injection of AITC depresses home cage wheel running in a concentration-dependent manner. **a** Administration of 10% AITC depressed wheel running for 3 h. The magnitude and duration of AITC-induced depression of wheel running was greatly reduced when the concentration was reduced to 1%. **b** Total percent of wheel revolutions during the 3 h following administration of 10% AITC (*n* = 17) was significantly lower compared to animals receiving 1% AITC (*n* = 20), mineral oil (*n* = 7), or saline (*n* = 14). *indicates *p* < 0.001 vs. saline, *p* = 0.016 vs. mineral oil, *p* = 0.04 vs. 1% AITC

Microinjection of AITC onto the dura caused a concentration-dependent reduction in wheel running. The highest concentration of AITC (10%) caused a pronounced depression of wheel running that lasted for 3 h (Fig. 1a). Administration of a lower concentration of AITC caused a more modest and shorter lasting depression of wheel running (Fig. 1a). Analysis of the magnitude of AITC-induced depression of wheel running during this 3-hour period (Fig. 1b) revealed a significant difference in wheel running between AITC conditions (*F*(3,173) = 7.459, *p* < 0.001). Post-hoc analysis revealed that wheel running was significantly lower following administration of 10% AITC compared to saline-, mineral oil-, or 1% AITC-treated rats (Tukey test: Sal vs. 10% AITC, *p* < 0.001; Mineral oil vs. 10% AITC, *p* = 0.016; 1% vs. 10% AITC, *p* = 0.04). The depression in wheel running following administration of 1% AITC was not significantly different than saline- (Tukey test, *p* = 0.153) or mineral oil-treated (Tukey test, *p* = 0.710) controls.

### Experiment 2: Repeated 10% AITC injections

**Fig. 2:**
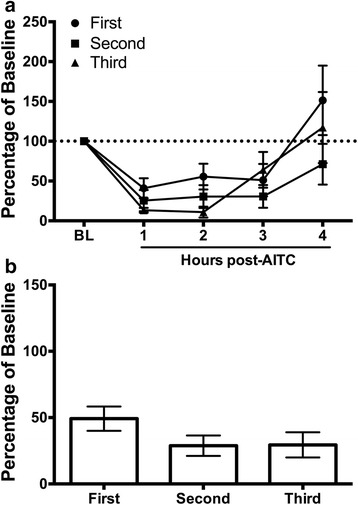
Consistent depression of wheel running following repeated injections of 10% AITC onto the dura. **a** Administration of 10% AITC depressed wheel running for approximately 3 h on all three trials. The magnitude of depressed wheel running was greatest following the third AITC injection, especially during the second hour, although this difference did not reach statistical significance when total wheel revolutions during the 3 h following AITC administration were analyzed (see Fig **b**). *n* = 6–7/condition

Rats were given three dural injections of 10% AITC to test the reliability of repeated injections to depress wheel running. Administration of 10% AITC caused a consistent depression of wheel running that lasted approximately 3 h following each injection (Fig. 2a). The magnitude of the depression seemed to increase with each subsequent injection, especially during the second hour, but, a one-way ANOVA on total wheel running during the 3 h following AITC administration revealed no significant differences between the three injections (*F*(2,56) = 1.716, *p* = .189; Fig. 2b).

### Experiment 3: Sumatriptan efficacy against AITC-induced pain

**Fig. 3:**
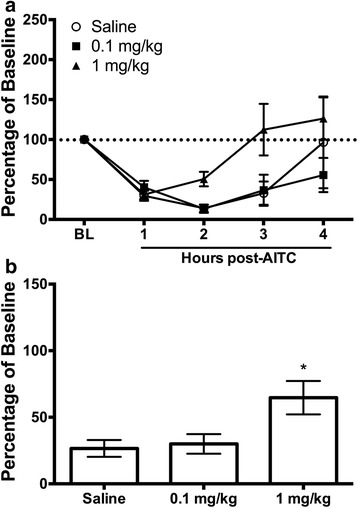
Administration of sumatriptan reverses AITC-induced depression of wheel running*.* **a** Administration of the high (1 mg/kg), but not the low dose (0.1 mg/kg) of sumatriptan immediately after administration of AITC shortened the duration of AITC-induced depression of wheel running. **b** Recovery of wheel running in the 3 h following sumatriptan (1 mg/kg; *n* = 11) administration was significantly greater following administration of 1 mg/kg of sumatriptan compared to rats treated with 0.1 mg/kg (*n* = 7) or saline (*n* = 7). *indicates *p* < 0.05 vs. saline

Administration of the anti-migraine medication sumatriptan immediately after injection of AITC attenuated the depression of wheel running. Reversal of AITC-induced depression of wheel running was first evident 2 h after sumatriptan administration and only following administration of the highest dose (1.0 vs. 0.1 mg/kg) (Fig. 3a). There was a significant difference in wheel running during the first three hours following AITC administration (Fig. 3b) in rats injected with 1 mg/kg of sumatriptan compared to rats treated with saline or a low dose of sumatriptan (0.1 mg/kg) (*F*(2,71) = 4.041, *p* = .022).

**Fig. 4:**
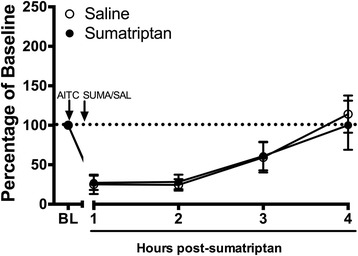
Sumatriptan injected 90 min after induction of headache did not reverse AITC-induced depression of wheel running. Sumatriptan (1 mg/kg) or saline (1 mL/kg) was injected 90 min after dural injection of 10% AITC. Administration of sumatriptan (*n* = 8) at this time point had no effect on depressed wheel running compared to saline-treated animals (*n* = 7)

A separate group of rats was injected with sumatriptan (1 mg/kg) or saline 90 min after AITC administration to determine whether sumatriptan could reverse migraine pain once it had been established. Running patterns are nearly identical between sumatriptan- and saline-treated rats when injected 90 min after AITC (Fig. 4). Comparison of total wheel running in these two groups during the 3 h following sumatriptan administration revealed no differences between groups (*t*(43) = 0.002, *p* = .969).

### Experiment 4: Sumatriptan effects on wheel running

**Fig. 5:**
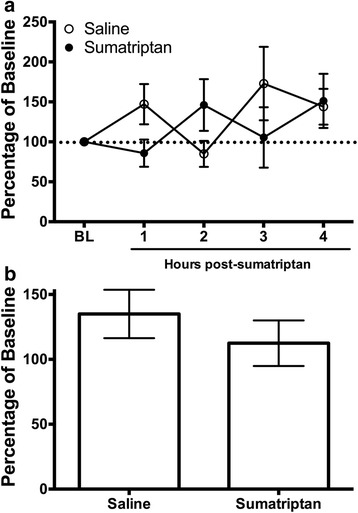
Sumatriptan had no effect on wheel running in rats not treated with AITC. **a** Wheel running was variable following administration of sumatriptan (1 mg/kg) or saline (1 mL/kg) in the absence of headache. Neither sumatriptan (*n* = 14) nor saline (*n* = 18) depressed wheel running. **b** Wheel running was unaffected in the 3 h following sumatriptan or saline

Naïve rats were injected with either sumatriptan (1 mg/kg) or saline to determine the effects of sumatriptan alone on wheel running. Sumatriptan had no consistent effect on wheel running in naïve animals (Fig. 5). Comparison of these two groups during the first 3 h after sumatriptan administration revealed no significant difference (*t*(94) = .860, *p* = .392).

## Discussion

The present data indicate that depression of home cage wheel running is a reliable and clinically relevant method to assess migraine pain in laboratory rats. AITC-induced activation of dural afferents produced a concentration-dependent reduction in wheel running that persisted for at least 3 h. Repeated injections of 10% AITC onto the dura caused a consistent reduction in wheel running following each injection. Finally, administration of the anti-migraine medication sumatriptan produced a dose-dependent reversal of AITC-induced depression of wheel running when administered immediately after the AITC injection, but did not affect wheel running in pain-free animals.

Our finding that dural administration of AITC produces a 3 h reduction in wheel running confirms previous studies indicating that activation of TRPA1 receptors produces migraine-like pain. TRPA1 receptors have been shown to contribute to migraine in both human and rat. Activation of trigeminal TRPA1 receptors causes meningeal vasodilation and calcitonin gene-related peptide release in rodents [21] and periorbital and hindpaw allodynia [9]. Case studies have reported that inhalation of TRPA1 agonists can trigger headache in people [3, 21].

Although the depression of wheel running following AITC administration onto the dura lasted for 3 h, the diagnostic criterion for migraine in humans requires a duration of a minimum of 4 h [12]. It is unlikely that AITC administration produces a syndrome that fully replicates a human migraine. Dural administration of an “inflammatory soup” has also been used to induce migraine-like pain, but neither of these models truly captures a human migraine. Given that migraine is a complicated process that involves the brain, local blood vessels, and trigeminal afferents [13], simple chemical activation of dural afferents via AITC or inflammatory soup is unlikely to engage all of these systems. However, the three-hour depression of wheel running reported in this manuscript and the widespread allodynia reported by others [9] suggests that administration of AITC mimics migraine-like pain.

Previous studies have shown that administration of AITC to the dura produces allodynia that persists for up to 5 h post-injection [9] - significantly longer than depression of wheel running. Given that allodynia may be present during interictal phases [1], it is likely that assessing allodynia as a measure of migraine in rodents may be confounded by allodynia being present in either the postdrome phase of a migraine attack or the interictal period. Furthermore, given that dural AITC-induced hindpaw allodynia persists for 5 h, it is unlikely that depression of wheel running is caused by hindpaw allodynia.

We also show that repeated AITC administration did not cause a significant increase in the magnitude or duration of depression of wheel running. A trend towards greater depression of wheel running was evident with repeated injections, particularly in the second hour post-AITC (Fig. 2a), but this difference did not reach statistical significance. A floor effect may have prevented the expression of more intense pain. Sensitization of the trigeminovascular system has been demonstrated using multiple dural infusions of inflammatory soup [20, 22] so a similar enhancement would be expected with repeated AITC injections. These findings suggest that it may be possible to induce chronic migraine with repeated dural injections of AITC. Continuous home cage wheel running would provide an objective and simple method to detect potential spontaneous migraines, especially if the incidence of chronic migraine arises in only a subset of animals as is the case with humans.

Sumatriptan is a prototypical anti-migraine agent and has been used to demonstrate the predictive validity of migraine models. Our data showing that sumatriptan is only effective in reversing AITC-induced depression of wheel running when given at a sufficient dose (1 mg/kg) and latency following induction of headache further validates home cage wheel running as a method to assess migraine in rats. Sumatriptan had no efficacy when administered 90 post-AITC injection (Fig. 4) as has been reported in humans where the efficacy of triptans is attenuated if patients wait to take the drug [8]. Furthermore, similar to the clinical situation [31], our data suggest that sumatriptan relieves pain 2 h after its administration (Fig. 3a). Although pre-treatment of sumatriptan may prevent AITC-induced depression of wheel running as has been reported previously with other behaviors [9, 20], triptans are generally considered abortive anti-migraine agents, and thus, are rarely taken before the onset of migraine symptoms.

Depression of wheel running is also a clinically relevant method to assess migraine pain in rats in that it mimics the reduction in normal physical activity characteristic of migraine in humans [12]. Migraine has been reported to reduce activity for 57.3 million days per year for women [26], and is often so severe as to require bed rest [4]. Our data showing depression of home cage wheel running is consistent with these clinical observations.

Assessment of home cage wheel running provides a number of advantages over other tests to assess migraine pain in animals. In contrast to pain-evoked methods to assess migraine such as assessing periorbital and/or hindpaw allodynia, which may induce stress by testing the animal in a novel environment, home cage wheel running reveals the effect of spontaneous migraine pain in a stress-free environment. Moreover, data collection is objective, independent of the researcher, and captures both the magnitude and duration of migraine pain.

Given that home cage wheel running is also sensitive to the disruptive side effects of analgesics [15], this test allows existing and experimental treatments to be simultaneously evaluated for both anti-migraine efficacy and side effect profile. This key advantage separates home cage wheel running from traditional tests of migraine pain in rats such as assessment of allodynia, which can be blocked by drugs with either antinociceptive or sedative effects. A key goal of drug treatment should be to restore function as opposed to replacing one problem (pain) with another (sedation).

Although depression of wheel running provides an objective and clinically relevant measure of migraine pain, exercise itself has been shown to reduce pain [11]. However, this type of exercise-induced antinociception typically requires multiple weeks of daily wheel running [24]. Our previous studies [14, 15] and those of others [30] show that 5–8 days of baseline wheel running has no direct antinociceptive effect. These findings are consistent with human behavior in that consistent prolonged exercise can be used to maintain health, whereas pain or illness will disrupt a person’s exercise regimen.

The present data build a firm foundation for the use of home cage wheel running to assess migraine pain in rats. The obvious problem with preclinical migraine research is that it does not mimic the spontaneous onset of migraine. Both pharmacological [23] and genetic [6] approaches have attempted to overcome this disconnect between preclinical models and the human situation. As before, these procedures have been limited because of the inability to continuously monitor migraine pain-related decreases in behavior. Assessment of home cage wheel running provides a simple method to detect such seemingly random occurrences of migraine in a subset of animals. The validity of home cage wheel running as a preclinical method to assess migraine will be tested in future studies by examining the effects of prophylactic and other abortive anti-migraine therapies.

Female rats were used in the present study to enhance the clinical relevance of the research given that migraine is more common in women than men [25, 26]. Given that the higher incidence of migraine in women has been linked to female sex hormones [29], one would predict that the magnitude and duration of migraine-induced depression of wheel running would be greater in female than male rats, and vary depending on the phase of the estrous cycle. However, the occurrence of sex differences may require the use of a more natural migraine model (e.g., systemic nitric oxide donor) as opposed to that induced by an irritant such as AITC or inflammatory soup.

## Conclusions

In sum, the results of the present study demonstrate that home cage wheel running objectively captures headache-induced depression of activity that closely resembles migraine pain in patients. It is an easy-to-use test that allows precise quantification of the magnitude and duration of migraine pain. The use of home cage wheel running should help unveil the pathophysiology underlying migraineheadache and provide a clinically relevant method to assess the anti-migraine efficacy of novel therapeutics.

## References

[1] Aguggia M (2012). Allodynia and migraine. Neurol Sci.

[2] Akerman S, Romero-Reyes M, Holland PR (2016) Current and novel insights into the neurophysiology of migraine and its implications for therapeutics. Pharmacol Ther. http://dx.doi.org/10.1016/j.pharmthera.2016.12.005 10.1016/j.pharmthera.2016.12.005 27919795

[3] Benemei S, Appendino G, Geppetti P (2010). Pleasant natural scent with unpleasant effects: cluster headache-like attacks triggered by Umbellularia californica. Cephalalgia.

[4] Brandes JL (2002). Global trends in migraine care: results from the MAZE survey. CNS Drugs.

[5] Burstein R, Collins B, Jakubowski M (2004). Defeating migraine pain with triptans: a race against the development of cutaneous allodynia. Ann Neurol.

[6] Chanda ML, Tuttle AH, Baran I, Atlin C, Guindi D, Hathaway G, Israelian N, Levenstadt J, Low D, Macrae L, O’Shea L, Silver A, Zendegui E, Mariette Lenselink A, Spijker S, Ferrari MD, van den Maagdenberg AMJM, Mogil JS (2013). Behavioral evidence for photophobia and stress-related ipsilateral head pain in transgenic Cacna1a mutant mice. Pain.

[7] Christensen SLT, Petersen S, Sørensen DB, Olesen J, Jansen-Olesen I (2016). Infusion of low dose glyceryl trinitrate has no consistent effect on burrowing behavior, running wheel activity and light sensitivity in female rats. J Pharmacol Toxicol Methods.

[8] Diener HC, Dodick DW, Goadsby PJ, Lipton RB, Almas M, Parsons B (2008). Identification of negative predictors of pain-free response to triptans: analysis of the eletriptan database. Cephalalgia.

[9] Edelmayer RM, Le LN, Yan J, Wei X, Nassini R, Materazzi S, Preti D, Appendino G, Geppetti P, Dodick DW, Vanderah TW, Porreca F, Dussor G (2012). Activation of TRPA1 on dural afferents: a potential mechanism of headache pain. Pain.

[10] Fischer L, Lavoranti MI, de Oliveira Borges M, Miksza AF, Sardi NF, Martynhak BJ, Tambeli CH, Parada CA (2016) TRPA1, substance P, histamine and 5-hydroxytryptamine interact in an interdependent way to induce nociception. Inflamm Res.:1–12. doi:10.1007/s00011-016-1015-1 10.1007/s00011-016-1015-1 27904941

[11] Grace PM, Fabisiak TJ, Green-Fulgham SM, Anderson ND, Strand KA, Kwilasz AJ, Galer EL, Walker FR, Greenwood BN, Maier SF, Fleshner M, Watkins LR (2016). Prior voluntary wheel running attenuates neuropathic pain. Pain.

[12] Headache Classification Committee of the International Headache Society (IHS) (2013). The International Classification of Headache Disorders, 3rd edition (beta version). Cephalalgia.

[13] Jacobs B, Dussor G (2016) Neurovascular contributions to migraine: Moving beyond vasodilation. Neurosci 338:130–144 10.1016/j.neuroscience.2016.06.012 PMC5083225 27312704

[14] Kandasamy R, Calsbeek JJ, Morgan MM (2016). Home cage wheel running is an objective and clinically relevant method to assess inflammatory pain in male and female rats. J Neurosci Methods.

[15] Kandasamy R, Calsbeek JJ, Morgan MM (2017) Analysis of inflammation-induced depression of home cage wheel running in rats reveals the difference between opioid antinociception and restoration of function. Behav Brain Res 317:502–507 10.1016/j.bbr.2016.10.024 PMC5107346 27746208

[16] Louter MA, Bosker JE, van Oosterhout WPJ, van Zwet EW, Zitman FG, Ferrari MD, Terwindt GM (2013). Cutaneous allodynia as a predictor of migraine chronification. Brain.

[17] Malick A, Jakubowski M, Elmquist JK, Saper CB, Burstein R (2001). A neurohistochemical blueprint for pain-induced loss of appetite. Proc Natl Acad Sci.

[18] Mannix S, Skalicky A, Buse DC, Desai P, Sapra S, Ortmeier B, Widnell K, Hareendran A (2016). Measuring the impact of migraine for evaluating outcomes of preventive treatments for migraine headaches. Health Qual Life Outcomes.

[19] Mathew NT, Kailasam J, Seifert T (2004). Clinical recognition of allodynia in migraine. Neurology.

[20] Melo-Carrillo A, Lopez-Avila A (2013). A chronic animal model of migraine, induced by repeated meningeal nociception, characterized by a behavioral and pharmacological approach. Cephalalgia.

[21] Nassini R, Materazzi S, Vriens J, Prenen J, Benemei S, De Siena G, la Marca G, Andrè E, Preti D, Avonto C, Sadofsky L, Di Marzo V, De Petrocellis L, Dussor G, Porreca F, Taglialatela-Scafati O, Appendino G, Nilius B, Geppetti P (2012). The “headache tree” via umbellulone and TRPA1 activates the trigeminovascular system. Brain.

[22] Oshinsky ML, Gomonchareonsiri S (2007). Episodic dural stimulation in awake rats: a model for recurrent headache. Headache.

[23] Oshinsky ML, Sanghvi MM, Maxwell CR, Gonzalez D, Spangenberg RJ, Cooper M, Silberstein SD (2012). Spontaneous trigeminal allodynia in rats: a model of primary headache. Headache.

[24] Sluka KA, O’Donnell JM, Danielson J, Rasmussen LA (2013). Regular physical activity prevents development of chronic pain and activation of central neurons. J Appl Physiol.

[25] Smitherman TA, Burch R, Sheikh H, Loder E (2013). The prevalence, impact, and treatment of migraine and severe headaches in the United States: a review of statistics from national surveillance studies. Headache.

[26] Stang PE, Osterhaus JT (1993). Impact of migraine in the United States: data from the National Health Interview Survey. Headache.

[27] Strassman AM, Burstein R (2013). A new animal model of headache: Ongoing pain vs stimulus-evoked hypersensitivity. Cephalalgia.

[28] Sufka KJ, Staszko SM, Johnson AP, Davis ME, Davis RE, Smitherman TA (2016). Clinically relevant behavioral endpoints in a recurrent nitroglycerin migraine model in rats. J Headache Pain.

[29] Vetvik KG, MacGregor EA (2016) Sex differences in the epidemiology, clinical features, and pathophysiology of migraine. Lancet Neurol 16:76–87 10.1016/S1474-4422(16)30293-9 27836433

[30] Whitehead RA, Lam NL, Sun MS, Sanchez J, Noor S, Vanderwall AG, Petersen TR, Martin HB, Milligan ED (2016). Chronic Sciatic Neuropathy in Rat Reduces Voluntary Wheel-Running Activity With Concurrent Chronic Mechanical Allodynia. Anesth Analg.

[31] Winner P, Mannix LK, Putnam DG, McNeal S, Kwong J, O’Quinn S, Richardson MS (2003). Pain-free results with sumatriptan taken at the first sign of migraine pain: 2 randomized, double-blind, placebo-controlled studies. Mayo Clin Proc.

